# The Pathways to the First Contact with Mental Health Services among Patients with Schizophrenia in Lagos, Nigeria

**DOI:** 10.1155/2013/769161

**Published:** 2013-12-31

**Authors:** Increase Ibukun Adeosun, Abosede Adekeji Adegbohun, Tomilola Adejoke Adewumi, Oyetayo O. Jeje

**Affiliations:** Federal Neuro-Psychiatric Hospital Yaba, 8 Harvey Road, PMB, Lagos 2008, Nigeria

## Abstract

There is increasing evidence that delay in the commencement of treatment, following the onset of schizophrenia, may be related to the pathways patients navigate before accessing mental health care. Therefore, insight into the pattern and correlates of pathways to mental care of patients with schizophrenia may inform interventions that could fast track their contact with mental health professionals and reduce the duration of untreated psychosis. This study assessed the pathways to mental health care among patients with schizophrenia (*n* = 138), at their first contact with mental health services at the Federal Neuro-Psychiatric Hospital Yaba Lagos, Nigeria. Traditional and religious healers were the first contact for the majority (69%) of the patients. Service users who first contacted nonorthodox healers made a greater number of contacts in the course of seeking help, eventuating in a longer duration of untreated psychosis (*P* < 0.001). However, the delay between the onset of psychosis and contact with the first point of care was shorter in patients who patronized nonorthodox practitioners. The findings suggest that collaboration between orthodox and nonorthodox health services could facilitate the contact of patients with schizophrenia with appropriate treatment, thereby reducing the duration of untreated psychosis. The need for public mental health education is also indicated.

## 1. **Introduction**

Schizophrenia is a severe, chronic, disabling mental disorder, with a lifetime morbid risk of about 1%, and a leading contributor to the global burden of diseases [1]. Despite the availability of effective therapy, there is a huge treatment gap for schizophrenia, with more than 40 million affected people in need of treatment in low and middle income countries [2–4]. Systematic reviews and meta-analytic studies have shown that delay in the commencement of appropriate treatment following the onset of psychosis is associated with more severe symptom profile, worse psychosocial functioning, poorer quality of life, and poorer treatment outcomes in patients with schizophrenia [5, 6]. Efforts at reducing the lag in the initiation of treatment for first episode schizophrenia has led to an increasing research interest in the pathways through which people with the disorders access care, with the view of identifying points of delay and, consequently, potential loci of interventions that could minimize the delay.

Pathways to care refer to the sequence of contacts an ailing person makes with services provided by individuals or organisations, prompted by the effort of the distressed persons and those of his or her significant others, in the process of seeking treatment for the ailment [7]. Sociocultural factors and health service variables such as the organisation, availability, and accessibility of health services exert influence on the pathways patients navigate in their route to care.

Nigeria is a low-middle income country, located in the western sub-Saharan Africa. It is the most populated country in the region with a population of about 150 million. Mental health services are disproportionately underresourced in Nigeria. There are 0.028 mental health outpatient facilities per 100,000 population and less than 200 psychiatrists [8]. The available mental health services are predominantly concentrated in large psychiatric hospitals and isolated from primary care in the community. Traditional health care system predates orthodox medical practice in Africa and continues to attract high patronage. The practice of traditional healers is consistent with traditional sociocultural belief systems about illness and its origin, and concepts about man's relationship with deities. Mental illness is traditionally perceived as arising from supernatural factors such as repercussion for offences against God, deities, and ancestors, or as a result of affliction from witchcraft and other preternatural forces.

A handful of studies have been conducted on the pathways to mental health care in Nigeria. Gureje et al. [9] studied the pathways of 159 patients with various psychiatric disorders presenting to a tertiary hospital in Ibadan, southwestern Nigeria. They found that traditional and religious healers were the first point of care consulted by the majority of the patients. Similarly, Abiodun [10] reported that about 40% of patients presenting to a tertiary mental health service in Ilorin, northcentral Nigeria, had previously contacted traditional or religious healers. A more recent study by Agara and Makanjuola [11] found that about 70% of patients have been treated by spiritual healers and 43% by traditional healers before presenting to a mental health facility in Akure, southwestern Nigeria.

Several studies have also been conducted on the pathways to mental health care in first episode schizophrenia or psychotic disorders, in other regions of the world [12, 13]. As previously highlighted in a systematic review of these studies [14], there are variations in the pathway to care across countries, which are attributable to differences in sociocultural, religious, and health service contexts. Physicians and other orthodox medical professionals or services are usually the first point of contact for patients with schizophrenia in western countries, whereas nonphysicians are the major first point of care for service users in Asia and Africa [9, 15–17]. The median number of previous pathway contacts before the patients accessed mental health services ranged from 1 to 4.5 across studies [15, 18].

Previous research regarding the determinants of pathway to care in first episode psychosis yielded inconsistent findings. Fuchs and Steinert [19] found that patients whose first contact in the pathway to care were nonphysicians had significantly longer duration of untreated psychosis. This finding was replicated by some other authors [20, 21]. On the other hand, other studies either found no significant association between duration of untreated psychosis (DUP) and pathways to care [22–24] or found longer treatment delays when service users made physicians or orthodox medical services their first contact in the pathway to care [15, 24]. Similarly, studies that determined the relationship between pathways to care in first episode psychosis and other patient's characteristics such as ethnicity [18, 25–27] gender [20, 25, 26, 28], socio-economic status [20, 25, 26, 29], and symptom profile [30–32] reported conflicting results.

Previous Nigerian studies on pathway to care examined heterogeneous samples of patients with various psychiatric disorders [9–11]. Studies have shown that pathways to care may vary across diagnostic categories, and especially, patients with psychotic disorders may differ from those with non-psychotic disorders in the route they navigate in their path to care [33, 34]. There is dearth of research on the pathways of patients with schizophrenia to mental health services in Africa. Therefore this study assessed the pathways to mental health care in patients with schizophrenia, at their first contact with a mental health service in Lagos, southwestern Nigeria. The association between the pathways to care and certain clinical and sociodemographic characteristics of the patients were also determined.

## 2. **Materials and Methods**

### 2.1. Study Location

The study was conducted at the Federal Neuro-Psychiatric Hospital, Yaba, Lagos, Nigeria. The Hospital is the largest psychiatric care facility in the country, with an in-patient facility of 535 beds and weekly outpatient clinic attendance of about 1,000 patients. Her clientele are drawn majorly from Lagos, a metropolis with a population of more than 10 million people, and neighbouring states in southwest Nigeria. The hospital has an open-door walk-in policy and there are no prerequisites for referrals or scheduling of appointments prior to uptake of patients.

### 2.2. Subjects

The sample consisted of new patients recruited on the day of their first presentation to the facility. Inclusion criteria consisted of (1) patients with a diagnosis of schizophrenia, according to the International Classification of Diseases (ICD-10) [35] diagnostic criteria, (2) patients whose presentation to the facility was the first contact with orthodox mental health care since onset of illness. Patients who were not accompanied by a relative, caregiver, or informant who had been acquainted with the patient before the onset of mental health problems were excluded. Data were obtained from patients and accompanying informants using a detailed semistructured questionnaire.

### 2.3. Procedure

Approval for the study was obtained from the Research and Ethical Committee of the Federal Neuro-Psychiatric Hospital, Yaba. Informed consent was obtained from patients and accompanying family members after the purpose and nature of the study were explained to them. Patients who met the inclusion criteria were consecutively recruited into the study after a comprehensive psychiatric evaluation and diagnosis by resident doctors in psychiatry, under the supervision of a consultant psychiatrist using the ICD-10 diagnostic criteria. The researchers were part of the clinical team that managed all the patients recruited into the study. The diagnoses of the patients were further ascertained with the Mini International Neuro-Psychiatric Interview (MINI) [36]. The symptom profiles of the patients were assessed with the Positive and Negative Syndrome Scale for Schizophrenia (PANSS) [37], an interviewer-based instrument. A semistructured questionnaire was used to elicit socio-demographic data, duration of illness/psychosis, and the pathways to care.

### 2.4. Measures

#### 2.4.1. Semistructured Questionnaire

This was designed by the authors to elicit data on the various modes of treatment or facilities patients consulted before presenting for professional mental health care in our facility. Based on data from the existing literature in Africa, participants were specifically probed about patronage of nonorthodox care such as religious and traditional healers including herbalist, prophets, and other Christian or Muslim clerics. Nonphysician pathways were defined as contacts to nonorthodox practitioners such as traditional or religious healers, while physician pathways consisted of contacts to general practitioners and other orthodox medical practitioners including psychiatrists. The duration of psychosis before consulting the first contact point and the reasons for the choice of contacts in the pathways to care were explored. The questionnaire also elicited socio-demographic data and the interval (in weeks) between the onset of psychotic symptoms and contact with professional mental health care. The latter was taken as a proxy measure of duration of untreated psychosis (DUP) since all the participants were commenced on antipsychotics at presentation. DUP is usually defined as the time from the manifestation of the first psychotic symptom to the initiation of antipsychotic drug treatment. The onset of psychotic symptoms was carefully reviewed and reconstructed with information provided by the patients and informants, with special attention paid on differentiating it from the onset of illness (emergence of first psychiatric symptom). A pretest on 20 subjects yielded satisfactory interrater reliability on the variables of interest (*r* = 0.88 to 0.92).

#### 2.4.2. Positive and Negative Syndrome Scale (PANSS) [37]

This was used to assess certain clinical characteristics in the patients with schizophrenia. It includes a structured interview to assess patients on 30 items covering positive and negative symptoms as well as general psychopathology. Of the thirty items included in the PANSS, seven constitute a positive scale, seven a negative scale, and the remaining sixteen a general psychopathology scale. For each item, ratings are made on a 1–7 scale of increasing levels of psychopathology ranging from absent to extreme. The scores for the scales are arrived at by summation of ratings for the component items. The instrument has been used by several authors in Nigeria.

#### 2.4.3. Mini International Neuropsychiatric Interview (MINI) English Version 5.0.0 [36]

This was used to ascertain the diagnosis of schizophrenia in the participants. The MINI was designed as a brief structured interview for the major Axis 1 diagnosis in the Diagnostic and Statistical Manual (DSM-IV) [38] and ICD-10. Validation and reliability studies done comprising the MINI to other similar structured interviews such as the structured clinical interview for the DSM-IV patient version (SCID-P) and the Composite International Diagnostic Interview [39] have shown high validity and reliability scores.

### 2.5. Statistical Analysis

(SPSS) Software (version 17) was used for the statistical analysis. The main outcome variable is the pathway to care of patients with schizophrenia which was dichotomised into nonphysician/nonorthodox and physician/orthodox pathways. Descriptive statistics such as frequencies, median, mean and standard deviation were generated for socio-demographic and clinical characteristics of the participants and other variables as appropriate. Relevant inferential statistics such as chi-square and *t*-test were used to determine the relationship between outcome and the variables assessed.

## 3. Results

The sample consisted of 138 patients with schizophrenia, at first presentation to a mental health service in Nigeria. The mean age of the participants was 36.29 (±11.12) years. Females constituted 60.1% of the sample (Table 1). The majority were unemployed and did not attain up to tertiary education.

The mean positive and negative scales scores of PANSS were (24.37 ± 7.86) and 14.05 (±8.81), respectively (Table 1). The mean general psychopathology scale score of the PANSS was 30.53 ± 9.24. The mean and median DUP were 101.18 (±162.45) weeks and 38 weeks, respectively.

The pathways to mental health care are highlighted in Table 2. The majority (69%) of the patients consulted spiritual or traditional healers as the first contact in the process of seeking care for the illness. Psychiatrists were the first contact for 17.4% of the patients, while 13.8% had previously consulted a nonpsychiatric physician or General Practitioner. At one time or the other during the course of the illness, 14.5% had sought help from both traditional and spiritual healers, 42.8% from spiritual healers alone, and 11.6% from traditional healers alone.

The delay between the onset of psychosis and contact with the first carer was shorter in patients patronising nonorthodox treatment. The first nonphysician contact was consulted within an average of 4 weeks after the onset of psychotic symptoms, whereas the first physician contact was made about 17 weeks after the onset of symptoms. There was significantly greater delay in initiating physician contact as compared with nonphysician contact (*P* < 0.001). Those who first consulted general practitioners made an average number of 0.98 (±0.5) care contacts before presenting to mental health professionals, while those whose first contact was traditional or religious healers consulted an average of 5.74 (±3.9) carers before presenting to mental health professionals.

The predominant reason accounting for the choice of spiritual and traditional healers rather than orthodox mental health service was the belief that the illness was spiritual or supernatural in origin (Table 2 and the Appendix).

Table 3 shows the association between the pathways to care and the sociodemographic and clinical characteristics of the patients. Patients with nonphysician contacts in their pathway to mental health services had longer duration of untreated psychosis (*P* < 0.001) and patronized a greater number of contacts (*P* < 0.001) in their pathways to care. There was no significant association between pathways to care and the PANSS scores of the participants. The PANSS scores of the participants are further highlighted according to their specific pathways to care (Table 4). Patients who first presented to General Practitioners and psychiatrists had marginally higher scores in the positive symptoms and general psychopathology subscales of the PANSS. On the other hand patients who first consulted traditional and spiritual healers had marginally higher negative symptoms subscale scores. The case summary in Appendix further highlights the interaction between sociocultural factors and the tortuous pathway to mental health care among the participants.

## 4. Discussion

To the best of our knowledge, this is the first prospective study of pathways to mental health care of patients with schizophrenia at first contact with mental health service in Nigeria. In contrast with findings from western populations in Europe [20, 29], USA [30], Canada [25], and Australia [40], nonphysicians (traditional and religious healers) were the first point of contact for the majority of patients with schizophrenia in the current sample. Similarly, patients with first episode psychosis in Asia commonly present to nonphysicians in the first instance [13, 15]. Our results are also consistent with findings of previous Nigerian studies on pathways to care among patients with other psychiatric disorders [9–11].

In African societies, the consultation of traditional and religious healers in the pathway to mental health care is best considered from the context of traditional viewpoints about the nature and origin of mental illness. Generally, diseases are regarded as spiritual phenomena determined by the interaction of vital forces including deities, ancestral spirits, living beings, animals, and objects [41, 42]. It is believed that subjects in the spiritual world can disrupt the functioning of the mind and cause mental illness [42]. According to this viewpoint, mental illness can be caused by the wrath of gods, malevolent human beings, witchcraft, sorcery, curses, and natural factors [42]. The natural factors include heredity, smoking, high fever, and “bad blood.” In many African communities, traditional healers existed before orthodox medicine, and their interventions are rooted in the sociocultural conception of illness. Traditional healers utilize divinations, consult oracles, and make sacrifices to appease the malevolent spirits or offended deities that cause mental illness. In southwest Nigeria, the god of divination (Ifa) is conceptualized as the god through whom healers can detect the cause of illness and the appropriate remedy for the illness. The practice of traditional healers also involves the use of scarification, potions derived from plant or animal extracts, and sacramental elements such as rituals, magical gestures, and symbolic objects.

In the current study, service users initiated contact with traditional/spiritual healers within one month after the onset of symptoms, while orthodox mental health services were not consulted until about nine months later. Furthermore, prior consultation with nonphysicians as compared with orthodox treatment resulted in a more tortuous pathway to mental health care. The patients who first consulted general practitioners presented to an average of about one carer before presenting to mental health professionals, while service users whose first contact was traditional or religious healers saw an average of about six carers before presenting to mental health professionals. These findings highlight the potential for collaboration between traditional and orthodox mental health practitioners. The traditional healers are based within their communities and are more accessible than orthodox medical services. There is scarcity of mental health professionals in Nigeria, and the majority of the services available for a population of 150 million are isolated in eight psychiatric hospitals and twelve University Hospitals, away from mainstream health care [43]. Orthodox mental health services are therefore inaccessible to the majority of the populace.

Delays in the initiation of appropriate treatment are associated with the exacerbation of psychotic symptoms, poor response to treatment, worse clinical outcomes, and poor quality of life in patients with schizophrenia. The DUP in the current study is higher than that reported in western populations. Previous studies reported mean and median DUP ranging from 22 to 150 weeks and 4.3–26 weeks, respectively [44, 45]. The association between the DUP and pathway to care is congruent with previous findings [5, 6]. Referral delays have been shown to contribute substantially to the duration of untreated psychosis [45]. Since patients with schizophrenia present early to traditional healers in Nigeria, there is a possibility of reducing the delay in accessing orthodox mental health service by the liaison of orthodox mental health professionals with traditional healers. Currently, there is no existing program on early intervention for psychosis in Nigeria. Early symptom detection and reduction in the delay of commencement of appropriate interventions by collaboration between the formal and informal healing sector are potentially feasible secondary prevention strategies in this regard.

There was no significant association between pathways to care and the socio-demographic characteristics of the participants. This highlights the preferential use of nonorthodox practitioners, regardless of level of education, age gender, and economic status. This finding suggests the need for universal public educational campaigns about the nature, aetiology, and availability of effective treatment for schizophrenia. Such interventions are promising in facilitating the early detection of psychosis, as highlighted in a recent systematic review of initiatives to reduce the duration of untreated psychosis [46].

In the current study, the positive symptoms and general psychopathology subscales PANSS scores of service users who first presented to General Practitioners and psychiatrists were marginally higher than those who first consulted traditional and spiritual healers. On the other hand, patients who first consulted traditional and spiritual healers had marginally higher negative symptoms subscale scores. This finding should be interpreted with caution due to the cross-sectional nature of the study and lack of data on the clinical profile of the patients at the onset of illness. However, some authors have reported that patients with positive symptoms such as delusions and hallucinations are more likely to make appropriate treatment contacts earlier in their pathway to care, while others differed in their findings. In a meta-analytic study of the relationship between DUP and symptom severity of schizophrenia at first treatment contact, Perkins et al. [6] demonstrated that longer DUP is associated with worse severity of negative symptoms. Therefore, the higher negative subscale score of patients who first consulted traditional or spiritual healers before presenting to orthodox care patients may be a reflection of their longer DUP. Further studies are needed to clarify the relationship between symptom profile and pathway to care in our cultural setting. Such data would provide useful information that could guide the planning of interventions.

This study has a number of limitations. The pathways to care and duration of untreated psychosis were not assessed by standardised validated measures. The extent to which recall bias and deliberate nondisclosure affected responses obtained from the participants is not known. Furthermore, the cross-sectional nature of the study precludes assertions about the impact of pathway to care on the symptom profile of the patients. However, this study provides a very vital data in a previously understudied population. Our methodology was similar to previous studies in order to facilitate comparability. The variables of interests were also clearly defined and understood by the respondents; a pre-test yielded satisfactory interrater reliability. Furthermore, the participants were prospectively assessed at first contact with mental health services.

## 5. Conclusion

In contrast to findings in western counties, patients with first episode schizophrenia in Nigeria first consult traditional and spiritual healers in their pathway to care, due to sociocultural viewpoints about mental illness. The delay between the onset of psychosis and contact with the first point of care was shorter in patients who patronized nonorthodox practitioners in the first instance. This finding suggests that, in the planning of mental health service delivery, consideration should be given to developing collaborations between orthodox and nonorthodox health systems. Such collaborations could facilitate earlier access to appropriate treatment and reduction in the duration of untreated psychosis. There is also a need for public mental health education about the nature and treatability of schizophrenia.

## Figures and Tables

**Table 1:** Sociodemographic and clinical characteristics of the participants.

*N* = 138	
Variable	*n* (%)
Age in years (mean ± SD)	36.29 ± 11.12
Sex	
Male	55 (39.9)
Female	83 (60.1)
Educational level	
Primary or Nil	25 (18.1)
Secondary	64 (46.4)
Tertiary	49 (35.5)
Employment status	
Employed	60 (43.5)
Unemployed	78 (56.5)
PANSS score:	
Positive scale score (mean ± SD)	24.37 ± 7.86
Negative scale score (mean ± SD)	14.05 ± 8.81
General psychopathology (mean ± SD)	30.53 ± 9.24
DUP in weeks (mean ± SD)	101.18 (±162.45)
DUP in weeks (median)	38.0

PANSS: positive and negative syndrome Scales; DUP: duration of untreated psychosis.

**Table 2:** Pathways to care and reasons for choosing nonorthodox^+^ healers.

Variable	*n* (%)
Pathways to care	
Spiritual	59 (42.8)
Traditional	16 (11.6)
GP/medical practitioner	19 (13.8)
Traditional and spiritual	20 (14.5)
Psychiatrist (our facility)	24 (17.4)
Reasons for choosing nonorthodox healers*	
Belief that the illness is due to supernatural factors	81 (85.3)
Influence of relatives, neighbours, or significant others	49 (51.6)
Stigma associated with illness	48 (50.5)
Ignorance about effectiveness of orthodox treatment	41 (43.2)
Lack of funds/poor access to orthodox care	33 (34.7)

GP: general practitioners.

*Total responses greater than 100% due to multiple responses.

^+^Nonorthodox refers to traditional or spiritual healers.

**Table 3:** Association between pathways to care and patients' characteristics.

Variables	Physician (orthodox)	Nonphysician (nonorthodox)	Statistics	*P*
	*N* = 43	*N* = 95		
	*n* (%)	*n* (%)		
Gender				
Male	16 (37.2)	39 (41.1)	*X* ^2^ = 0.18	0.669
Female	27 (62.8)	56 (58.9)		
Employment status				
Employed	23 (53.5)	37 (38.9)	*X* ^2^ = 2.55	0.111
Unemployed	20 (46.5)	58 (61.1)		
Educational level				
Tertiary	15 (34.9)	34 (35.8)	*X* ^2^ = 0.01	0.918
Secondary or less	28 (65.1)	61 (64.2)		
DUP*				
Short (≤38 weeks)	37 (86.0)	32 (33.7)	*X* ^2^ = 32.47	**<0.001**
Long (>38 weeks)	6 (14.0)	63 (66.3)		
Age in years (mean ± SD)	35.47 (±10.2)	37.24 (±11.5)	*t* = 0.87	0.387
Number of contacts^+^ (mean ± SD)	0.98 (±1.5)	5.74 (± 3.9)	*t* = 7.67	**<0.001**
PANSS				
Positive symptom (mean ± SD)	25.93 (±6.9)	23.67 (±8.2)	*t* = −1.53	0.127
Negative symptom (mean ± SD)	13.37 (±7.7)	14.37 (±9.3)	*t* = 0.60	0.548
General psychopathology (mean ± SD)	31.66 (±8.8)	30.02 (±9.4)	*t* = −0.90	0.368

*Duration of untreated psychosis was dichotomised at median split; ^+^number of contacts in the pathway to care before presenting to our facility.

The bold values are the significant *P* values (*P* < 0.005).

**Table 4:** The PANSS score of participants according to their specific pathways to care.

Pathway to care	Panss score		
	Positive symptom scale	Negative symptom scale	General psychopathology scale
Spiritual	23.56 ± 7.67	14.11 ± 9.04	26.62 ± 9.23
Traditional	20.38 ± 9.00	15.20 ± 9.68	28.88 ± 10.73
GP	28.28 ± 5.94	12.63 ± 4.65	29.00 ± 6.75
Trado-spiritual	24.09 ± 7.27	14.50 ± 10.35	25.17 ± 9.00
Psychiatrist	26.79 ± 8.18	12.59 ± 8.34	30.53 ± 9.24

## Appendix

### Case Summary

A. J. is a 24-year old single unemployed male, secondary school leaver who presented to our facility in the company of his mother and a neighbour. The patient is the only son of her mother. His father had two wives and lives in a different city. Presenting complaints included 2-year history of undue suspiciousness, false beliefs that his mother poisoned his meals, hearing voices of unseen people in clear consciousness, talking to self, and neglect of personal hygiene. Mental state examination revealed a poorly groomed man with scarification marks and on the face and limbs. His affect was constricted and inappropriate. He had persecutory delusion, delusion of control, third person auditory hallucinations, and lacked insight.

Enquiries about the pathway to care revealed that after the onset of the symptoms, A. J. was first taken to a traditional healer in a nearby village. The mother believed the abnormal behaviours exhibited by A. J. were due to the handiwork of his step-mother who had cast a spell on him. At the traditional healer's home, some incantations were made. A. J. was physically restrained with chains and treated with several local herbal concoction and scarification. However, there was no significant improvement in his condition six months after the commencement of treatment. Subsequently some neighbours advised her to seek for help with another herbalist reputed to have superior powers. He was admitted for another 4 months and appeared to make some improvement gradually.

Within a week of his discharge from the traditional home, symptoms became exacerbated. Thereafter, A. J. was referred to a mountain to see a spiritualist in a neighbouring town. Special prayers, vigils, and “deliverance sessions” were conducted for him. He was also given “holy water” to drink. He remained there for about three months before he returned home. He also patronised a couple of churches thereafter without any significant improvement. Subsequently they resigned to fate until a distant relative who knew someone who had been treated at our facility advised them to seek orthodox care at our facility.

A. J.'s mother reported that she knew about our facility, but they did not believe that drugs can treat mental illness, since it is “spiritual” in origin. She also believed that she would not be able to afford the treatment. Furthermore, the hospital was too far away from where she lives.

A diagnosis of schizophrenia was made and appropriate management commenced.
